# Efficacy of a radiofrequency thermocoagulation strategy targeting the propagation network in MRI-negative post-encephalitic insular epilepsy: a case report

**DOI:** 10.1007/s00701-025-06652-y

**Published:** 2025-08-27

**Authors:** Tomotaka Ishizaki, Satoshi Maesawa, Shun Yamamoto, Takahiro Suzuki, Hajime Hamasaki, Takafumi Tanei, Ryuta Saito

**Affiliations:** 1 Department of Neurosurgery, Nagoya University Graduate School of Medicine, Nagoya, Japan; 2 Epilepsy Center, Nagoya University Hospital, Nagoya, Japan; 3 Brain and Mind Research Center, Nagoya University, Nagoya, Japan; 4 Department of Neurosurgery, Nagoya Medical Center, Nagoya, Japan

**Keywords:** Claustrum, Epileptogenic network, Propagation network disconnection, Stereoelectroencephalography, Case report

## Abstract

Insular epilepsy after encephalitis is often drug-resistant and MRI-negative, limiting resection due to eloquent cortex involvement. We describe a case in which radiofrequency thermocoagulation (RFTC) was applied to disconnect the propagation network (PN) identified by stereoelectroencephalography. In a woman with focal to bilateral tonic–clonic seizures, the epileptogenic network (EN) was in the left insula and temporal operculum, and the PN spread to the perirolandic area. PN-targeted RFTC, guided by tractography, preserved the EN. At 18 months, seizures decreased by 95.6% with preserved function. Selective PN disconnection may be an option when EN resection is limited.

## Introduction

Post-encephalitic epilepsy is often drug resistant, and localizing the epileptic focus is challenging in magnetic resonance imaging (MRI)-negative cases. The epileptic network comprises the epileptogenic network (EN) and propagation network (PN), which are responsible for seizure onset and spread, respectively [1]. Accordingly, surgical strategies include EN disruption, PN disconnection, and neuromodulation. Advances in stereoelectroencephalography (SEEG)-based network evaluation have improved surgical planning.

We report a case of drug-resistant insular epilepsy following encephalitis, with no lesions detected on MRI or functional imaging. Instead of resecting or ablating the insula (EN disruption), we applied radiofrequency thermocoagulation (RFTC) to disconnect the PN between the insula and perirolandic area, achieving a favorable outcome.

### Case presentation

This research was approved by the appropriate ethics committee. Informed written consent was obtained from the patient. All methods were performed in accordance with the relevant guidelines and regulations.

**Fig. 1:**
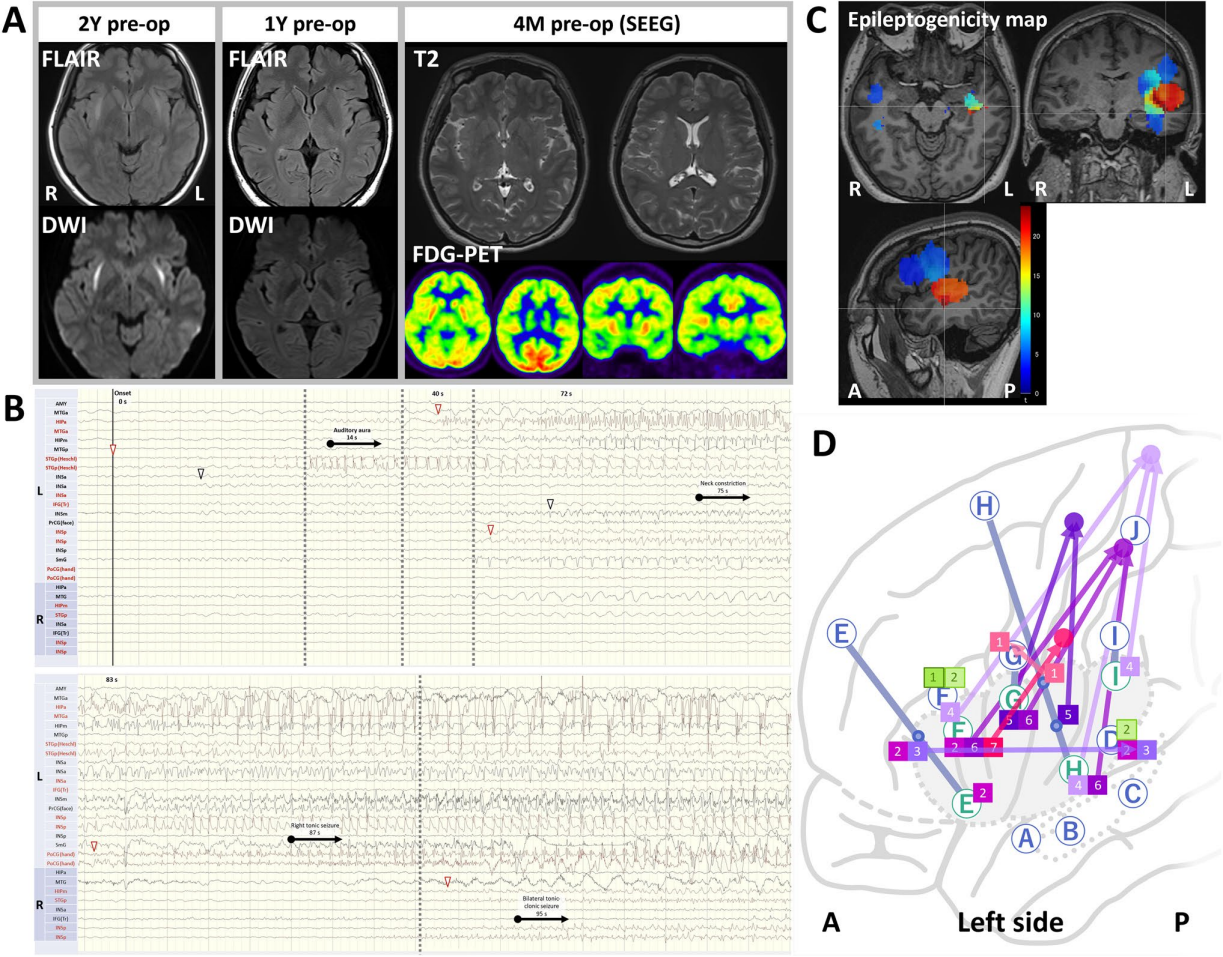
Preoperative imaging, SEEG findings, and cortical stimulation mapping for focus identification. (**A**) Preoperative magnetic resonance imaging (MRI) and fluorodeoxyglucose-positron emission tomography (FDG-PET) performed 2 years, 1 year, and 4 months (stereoelectroencephalography [SEEG] performed) prior to radiofrequency thermocoagulation. At encephalitis onset (2 years prior to surgery), MRI reveals hyperintensities in the bilateral claustrum on diffusion-weighted imaging and fluid-attenuated inversion recovery (arrowheads), which resolved before surgery. No lesions are identified on MRI or FDG-PET 4 months before surgery. (**B**) Ictal SEEG findings. Seizures originated in the left Heschl’s gyrus (arrowhead), followed by EEG changes in the anterior insula (INSa) and subsequent spread to the frontal and parietal lobes. (**C**) Epileptogenicity map. High epileptogenicity index (EI) values are localized from the left insula to the temporal operculum. (**D**) Cortical stimulation mapping across the left frontal, temporal, and parietal lobes. Letters indicate electrode sites. Green boxes indicate stimulation-induced neurological symptoms (1: phonemic paraphasia; 2: word-finding difficulty). Pink and purple boxes denote stimulation-induced ictal symptoms (1: rightward deviation due to tongue stiffness; 2: nausea indicating autonomic seizure; 3: auditory aura; 4: numbness in the right foot; 5: right upper limb tonic seizure; 6: numbness in the right upper limb; 7: numbness of the tongue). Arrows link stimulation sites to anatomically expected symptom locations. Abbreviations: *AMY*, amygdala; *MTGa*, anterior middle temporal gyrus; *MTGp*, posterior middle temporal gyrus; *HIPa*, anterior hippocampus; *HIPm*, middle hippocampus; *STGp*, posterior superior temporal gyrus; *STGp (Heschl)*, posterior superior temporal gyrus (Heschl’s gyrus); *INSa*, anterior insula; *INSm*, middle insula; *INSp*, posterior insula; *IFG (Tr)*, inferior frontal gyrus (triangular part); *PrCG* (face), precentral gyrus (face area); *PoCG* (hand), postcentral gyrus (hand area); *SmG*, supramarginal gyrus

A woman in her 20 s developed encephalitis of unknown etiology, presenting with fever. MRI revealed bilateral claustrum hyperintensities on diffusion-weighted imaging (DWI) and fluid-attenuated inversion recovery (FLAIR) (Fig. 1A). She fully recovered following steroid pulse therapy, and the lesions resolved 1 year preoperatively. However, she continued to experience focal to bilateral tonic–clonic seizures (FBTCS) daily, preceded by auditory aura, neck constriction, and right-sided numbness. This was followed by tonic–clonic movements of the right hand, face, and leg, progressing bilaterally. MRI (FLAIR, DWI) performed 1 year preoperatively and T2-weighted and fluorodeoxyglucose positron emission tomography conducted 4 months preoperatively showed no abnormalities (Fig. 1A). Video-electroencephalography (VEEG) captured multiple habitual seizures, with interictal spikes and ictal rhythmic activity in the left temporal and frontal regions. Left insular epilepsy was suspected, and SEEG was performed 4 months preoperatively.

SEEG recorded seizures consistent with the patient’s habitual seizures. Visual inspection of the SEEG ictal recordings revealed that the ictal activity originated in the Heschl’s gyrus, spreading to the anterior insula and then rapidly beyond the temporal and parietal opercula, with propagation to the perirolandic area (Fig. 1B). We calculated the epileptogenicity index (EI) values on the epileptogenicity map[1] based on the SEEG ictal recordings, and the results demonstrated that the highest EI values were localized to the left insular cortex and the adjacent temporal operculum (Fig. 1C). Cortical stimulation mapping[2] elicited language disturbances in the left inferior frontal gyrus. Insula stimulation induced multiple neurological symptoms not normally associated with insular function but corresponded to the habitual ictal symptoms. Anterior insula stimulation provoked auditory auras and numbness of the tongue. Stimulation across the entire insula elicited numbness in the right hand and foot, while that near the central sulcus of the insula triggered tonic seizures of the right hand. These findings suggested that the insula had formed an abnormal, hyperexcitable network linking the temporal operculum and the perirolandic area (Fig. 1D).

**Fig. 2:**
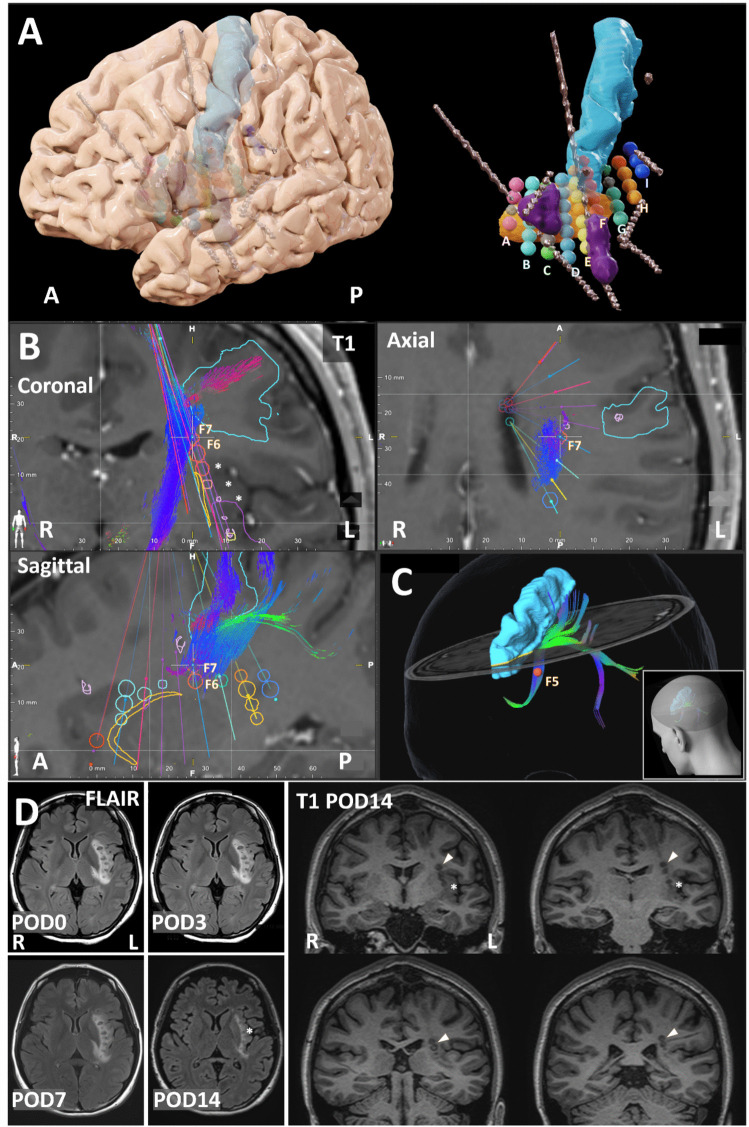
Presurgical planning and postoperative evaluation for RFTC. (**A**) Presurgical 3D planning of radiofrequency thermocoagulation (RFTC) lesions along trajectories A–I. Large spheres indicate 5-mm lesions, smaller spheres indicate 4-mm lesions, and gray spheres denote sites spared to avoid vascular injury. Light blue, precentral gyrus; orange, claustrum; purple, regions with elevated epileptogenicity index (EI) values. Metallic objects represent stereoelectroencephalography (SEEG) electrodes. (**B**) Fusion image of T1-enhanced magnetic resonance imaging (MRI) with presurgical 3D planning objects and diffusion tensor imaging (DTI)-based tractography. Color codes are consistent with (A). Orange-colored lesions F6 and F7 (trajectory F) are near the pyramidal tract; lesion F7 was reduced to 4 mm to avoid tract injury. Coagulation targeted the white matter between the claustrum (orange) and insula (asterisk). (**C**) White matter tracts included in lesion F5, expected to be disconnected by thermocoagulation. (**D**) Postoperative MRI (fluid-attenuated inversion recovery and T1 images). Edema around lesions subsided by postoperative day (POD) 14. The insular cortex (asterisk) is preserved, with ablation confined to deep white matter (arrowhead) adjacent to the pyramidal tract

Based on the SEEG findings, the EN was localized to the temporal operculum and insula, while the PN spread extensively to the perirolandic area via subcortical white matter tracts. Resection or thermocoagulation of all these cortical areas was not feasible. Furthermore, because the left hemisphere was language dominant, resection or ablation of the temporal operculum was functionally disadvantageous. Thus, we planned a disconnection strategy using RFTC directed at the subinsular claustrum and associated white matter tracts (Fig. 2A). Targeted tracts for each lesion were delineated during the planning phase by visualizing the specific fiber tracts to be targeted using diffusion tensor imaging tractography. We confirmed that the targeted tracts projected from the insula toward the perirolandic area. The placement of each coagulation site was accordingly planned to avoid injury to the pyramidal tract and perforating arteries (Fig. 2B and C). RFTC was performed under local anesthesia while monitoring for neurological symptoms. Simulation and coagulation were performed using a Leksell neurogenerator with a monopolar probe (1-mm diameter, 2-mm conductivity length; Elekta Instruments AB, Sweden). The probes were inserted stereotactically using a Leksell frame and a stereotactic robot (Neuromate; Renishaw, UK). Test stimulation (1–3 mA, 50 Hz, 500 µs) was performed at the planned lesion sites. In total, nine trajectories were implanted according to the preoperative plan, resulting in 44 coagulation lesions(40 lesions, 5-mm diameter, 70 °C, 60 s; 4 lesions, 4-mm diameter, 60 °C, 50 s) (Fig. 2D). Postoperative complications included transient, mild right lower-limb paresis and impaired fine motor function of the right hand, both of which resolved within 1 week. The patient was discharged 2 weeks postoperatively. At 18 months, auditory auras persisted 3–5 times per week, but FBTCS frequency reduced by 95.6% to only 2 per month.

## Discussion

The patient developed encephalitis of unknown etiology, with bilateral FLAIR hyperintensities observed in the claustrum during the acute phase. The clinical course and imaging findings suggested autoimmune encephalitis or febrile infection-related epilepsy syndrome [4]. However, despite an extensive systemic evaluation, including tests for anti-neuronal nuclear antibodies, no specific autoimmune markers were identified. Ictal SEEG findings showed that the seizures originated either simultaneously or in rapid succession from the left insula and temporal operculum (Heschl’s gyrus) and propagated to the frontal and parietal lobes. These findings supported the presence of a widespread EN encompassing the insula and operculum and a PN extending toward the perirolandic area. As the EN involved both the insular cortex and temporal operculum, RFTC targeting these areas was not feasible due to anatomical inaccessibility and eloquent cortex involvement in the language-dominant hemisphere. In addition, both the EN and PN in this case were widely distributed, and the spatial extent of the target exceeded the coverage achievable by RFTC via SEEG electrode. To our knowledge, PN-targeted RFTC for insular epilepsy has rarely been documented in the literature.

Surgical treatment for insular epilepsy has mainly been directed at removing or ablating the epileptic focus [3]. However, when the EN is widespread and involves the eloquent cortex, direct intervention targeting the focus becomes anatomically and functionally challenging; targeting the PN may be more effective [2]. Accordingly, instead of disrupting the EN within the insular cortex and temporal operculum, we disconnected the PN by applying RFTC to the propagation pathway composed of the claustrum and adjacent white matter fibers extending toward the perirolandic area. The claustrum was targeted because it functioned as a key gray matter relay for neural fibers originating from the insula and was readily identifiable on MRI. This enabled disconnection of the widespread PN, resulting in substantially reduced seizure frequency without compromising eloquent cortical function.

The auditory aura originating from Heschl’s gyrus typically persists but remains isolated and rarely progresses to FBTCS. These observations lend support to the hypothesis that disconnecting the PN while preserving the EN may reduce the likelihood of progression to FBTCS.

## Limitations

This report describes a single, uncontrolled case with 18 months of follow-up; thus, the generalizability and long-term durability remain uncertain. PN pathways were inferred from SEEG sampling and DTI tractography, both subject to spatial-resolution and sampling limitations. Complete PN disconnection was not feasible owing to proximity to perforating arteries and the pyramidal tract, leaving potential residual pathways. The effects of medication and natural fluctuations cannot be fully excluded.

## Conclusions

In MRI-negative post-encephalitic epilepsy, detailed analysis of ictal semiology combined with SEEG allows the identification of an EN involving the insula and temporal operculum, as well as a PN extending through the claustrum and subcortical white matter tracts. Selective focal disconnection of the PN using RFTC resulted in substantial seizure reduction without apparent compromise of the eloquent area. This case highlights the feasibility, safety, and potential effectiveness of an RFTC strategy targeting PN components (i.e., claustrum and associated white matter tracts) rather than the EN for insular epilepsy. When the EN involves eloquent or surgically challenging cortex, preserving the EN while selectively disconnecting the PN may represent a practical and pathophysiologically informed surgical alternative to EN resection.

## Data Availability

No datasets were generated or analysed during the current study.
